# Metabolomic Analysis of Pediatric Patients with Idiosyncratic Drug-Induced Liver Injury According to the Updated RUCAM

**DOI:** 10.3390/ijms241713562

**Published:** 2023-09-01

**Authors:** Francisco Andújar-Vera, María Luisa Alés-Palmer, Paloma Muñoz-de-Rueda, Iván Iglesias-Baena, Esther Ocete-Hita

**Affiliations:** 1 Bioinformatic Unit, Biosanitary Research Institute ibs.GRANADA, 18012 Granada, Spain; fandujar@ibsgranada.es; 2 Department of Pediatrics, University of Granada, 18016 Granada, Spain; estherocete@ugr.es; 3 Department of Pediatrics, “Virgen de las Nieves” University Hospital, 18014 Granada, Spain; 4 Research Support Unit, Biosanitary Research Institute ibs.GRANADA, 18012 Granada, Spain; palomalancha@ibsgranada.es; 5 Research Unit, GenActive Clinic & Research, 18193 Granada, Spain; genactive.info@gmail.com; 6 Biosanitary Research Institute ibs.GRANADA, 18012 Granada, Spain

**Keywords:** children, DILI, metabolomics, bile acids, liver injury, updated RUCAM

## Abstract

Hepatotoxicity, a common adverse drug effect, has been extensively studied in adult patients. However, it is equally important to investigate this condition in pediatric patients to develop personalized treatment strategies for children. This study aimed to identify plasma biomarkers that characterize hepatotoxicity in pediatric patients through an observational case–control study. Metabolomic analysis was conducted on 55 pediatric patients with xenobiotic liver toxicity and 88 healthy controls. The results revealed clear differences between the two groups. Several metabolites, including hydroxydecanoylcarnitine, octanoylcarnitine, lysophosphatidylcholine, glycocholic acid, and taurocholic acid, were identified as potential biomarkers (area under the curve: 0.817; 95% confidence interval: 0.696–0.913). Pathway analysis indicated involvement of primary bile acid biosynthesis and the metabolism of taurine and hypotaurine (*p* < 0.05). The findings from untargeted metabolomic analysis demonstrated an increase in bile acids in children with hepatotoxicity. The accumulation of cytotoxic bile acids should be further investigated to elucidate the role of these metabolites in drug-induced liver injury.

## 1. Introduction

Hepatotoxicity or drug-induced liver injury (DILI) is defined as liver injury or damage caused by exposure to a drug or other chemical agent that is not part of the composition of living organisms. DILI can be intrinsic when the substance, above a certain dose, has the capacity to damage the liver. This is a predictable form of toxicity that occurs in most mammals and, therefore, can be reproduced in experimental animals. This form, however, is very rare; the prototype drug that produces this type of lesion is paracetamol. Idiosyncratic toxicity is more common. This is a complex, multifactorial disease that may involve factors associated with the poison, the patient, and/or the environment. They are not predictable and are species-specific; thus, they cannot be reproduced. As a consequence, the patient is the sole source of the study. This toxicity, in turn, is divided into metabolic, when the damage is due to the direct effect of the drug or to its active metabolite, and immunoallergic, when the damage is mediated by the immune system. This division is theoretical since both mechanisms are usually involved, although at different levels of intensity [1,2].

Childhood is characterized by growth and development, with a gradual process of organ and system maturation and consequent variability in drug kinetics and response [3]. This circumstance makes the child especially vulnerable to the adverse effects of medications. However, the scientifically valid information on which prescriptions for children are currently based is very scarce. In many cases, the information used is that available for the adult population, since clinical trials in children are difficult to perform [4,5].

In pediatric medicine, DILI is a rare clinical situation. However, it has high morbidity and mortality, and it is one of the most common causes of acute liver failure. Furthermore, its diagnosis is clinically challenging due to its low incidence and phenotypic variability [6,7,8].

According to Ocete-Hita et al. [9], the incidence of adverse drug reactions in children is 15.1 per 1000 children. This represents 2% of admissions to a pediatric hospital, which is similar to the rate observed for adult patients. Fewer than 8% of these children may present liver involvement, which can range from a slight increase in transaminases to fulminant hepatitis. However, the true value may be higher, due to incomplete notifications by clinicians or, as mentioned above, the absence of precise diagnostic methods, since this continues to be a diagnosis of exclusion, which requires not only the patient’s medical history, but also laboratory tests and, occasionally, a liver biopsy [10].

Ye et al. (2021), in a 10-year retrospective study that included 77 newborns and 261 children, in which causality was evaluated using the RUCAM (Roussel Uclaf Causality Assessment Method) method, medium- and long-chain fat emulsions and sodium glycerophosphate were strongly associated with DILI in newborns, while omeprazole and methylprednisolone sodium succinate played a significant role in DILI in children [5].

There is currently great interest in enhancing our understanding of DILI, from clinical, epidemiological, and molecular standpoints, as this condition has a major impact on patients, drug development and healthcare costs.

Although numerous theories for drug-induced liver injury (DILI) have been suggested, a definitive causal link among drugs, risk elements, and DILI mechanisms has yet to be established. The optimal diagnostic strategy consists of a blend of strong clinical suspicion, a thorough medical history including risk factors and their timeline, and detailed hepatological examinations. These are guided by the standards of the internationally recognized Roussel Uclaf Causality Assessment Method, which is considered a key diagnostic tool for DILI [11].

In the context of pharmacogenomics associated with drug-induced liver injury (DILI), it serves not only to identify novel optimal biomarkers but also to augment the accuracy of differential diagnosis pertaining to subclinical DILI. Nevertheless, the tangible utilization of pertinent genetic testing in clinical practice currently encounters constraints, as the infrequent occurrence of DILI invariably results in a diminished positive predictive value (PPV) for the discerned genetic variations. Despite these challenges, this avenue warrants further exploration in future investigations [12].

Metabolomics is widely recognized as a useful phenotyping tool for revealing dysregulated metabolic pathways in cells and tissues, thus facilitating the analysis of disease and treatment responses. The metabolome provides a direct global readout of the dynamic biochemical state of a biological system, and it is increasingly applied to the study of liver diseases, such as xenobiotic hepatotoxicity [13,14,15]. Although some preliminary exploratory research has been conducted [16,17,18], little use has been made of this technique in the study of hepatotoxicity in adults, with no studies in children.

Assuming that the hepatocyte endometabolome is likely to be mirrored to some extent by the hepatic exometabolome, our aim in this study was to identify metabolic changes in the serum of pediatric DILI patients that reflect the differential features of childhood hepatotoxicity.

The working hypothesis of the current study was that metabolomics could serve as a potent instrument for the identification of biomarkers that elucidate dysregulated metabolic pathways in pediatric drug-induced liver injury (DILI). 

This investigation represents the inaugural endeavor in exploring the metabolomic profile in pediatric patients who have experienced a DILI episode.

## 2. Results

### 2.1. Data Matrix and Analytical Validation

The initial matrix of variables consisted of 1710 extracted signals; however, only the monoisotopic ones were selected, which reduced this matrix to 670 mass/charge. In the next stage of the process, the signal of the quality samples was compared with that found in the sample prepared as a blank; those signals that appeared in the blanks were eliminated from the analysis, thus producing an array of 373 variables. Finally, the coefficient of variation of all the signals in the quality control samples was calculated, to detect those presenting initial differences during the analysis. In this step, another 12 signals were eliminated, giving us a final matrix with 361 variables, on which the statistical analysis was performed.

**Figure 1:**
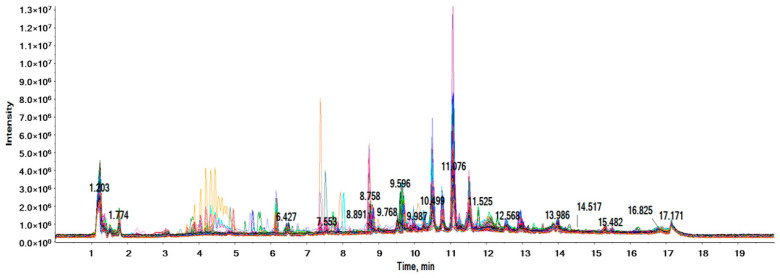
Total ion current for all plasma samples included in the analysis.

Figure 1 shows the total ion current obtained for all the samples considered.

As can be seen in the chromatogram, the analysis was highly reproducible, since the overlapping of all the signals was perfectly apparent, indicating little to no variability in the retention time drift by which the different metabolites could be detected. This finding was corroborated by reviewing different signals throughout the chromatogram and evaluating the changes in retention times; in no case did the metabolites drift from one sample to another by more than 0.1 min.

**Figure 2:**
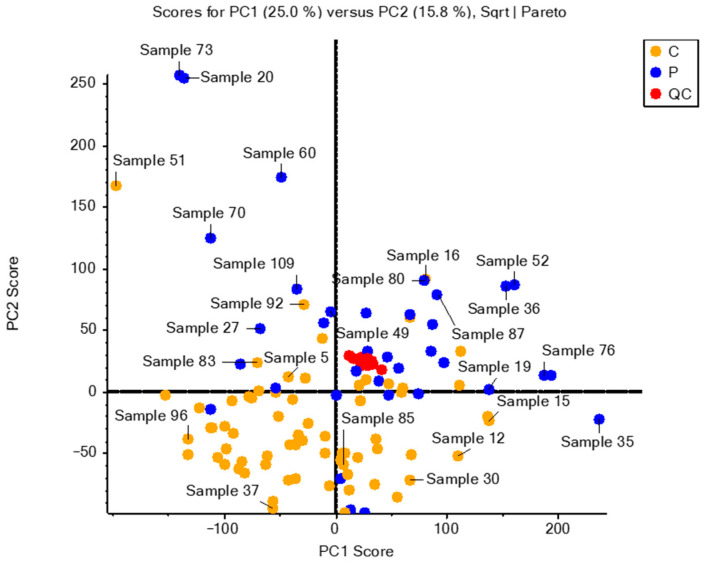
Principal component analysis for all samples included in the analysis. Principal components 1 and 2 (PC1 and PC2) representing the most variation and the second most variation in the data, respectively. Red circles correspond to quality control (QC) samples, orange circles correspond to samples from control subjects (C), and blue circles correspond to samples from children with hepatotoxicity (P).

The analytical validation was conducted using PCA, an unsupervised method, considering the 361 variables included in the statistical analysis (see Figure 2).

Figure 2 shows a perfect grouping of the QC samples, thus validating the analysis in terms of reproducibility in the injections. Furthermore, two distinct clusters can be observed among the groups included in the study, indicating significant differences in metabolomic composition within these groups.

### 2.2. Statistical Analysis

**Figure 3:**
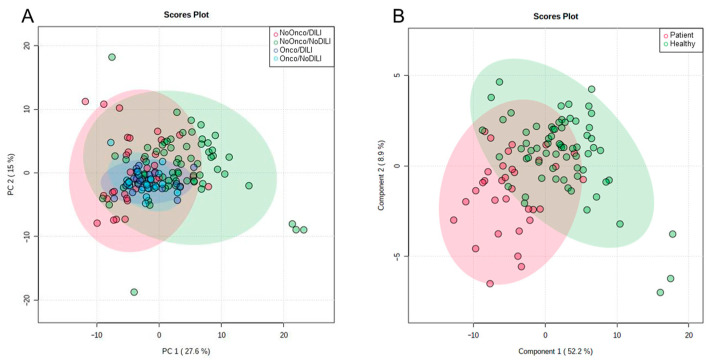
Multivariate analysis using unsupervised and supervised methods. (**A**) Principal component analysis for the four groups. Red circles: non-oncological children with hepatotoxicity. Green circles: non-oncological children without hepatotoxicity. Dark-blue circles: oncological children with hepatotoxicity. Light-blue circles: oncological children without hepatotoxicity. (**B**) Regression by partial least squares for the two groups. Green circles: controls. Red circles: children with hepatotoxicity.

The unsupervised multivariate analysis revealed the existence of a group of cancer patients, some in the control group and others in the DILI group. These children presented similar and differential characteristics, very probably due to the cancer, and were excluded from the analysis because this circumstance might have interfered with the study aim, i.e., to detect specific biomarkers of liver toxicity (see Figure 3A). Therefore, the study was completed with 32 children with DILI and 66 controls. Clinical presentation and characteristics of the 32 children with DILI are summarized in Table 1.

The 361-variable matrix was processed using MetaboAnalyst 5.0 (RRID:SCR_015539) as follows. The data were normalized using a reference quality control, transformed using the logarithmic function, and scaled using the autoscaling model. This matrix contained, for subsequent analysis, 32 children with DILI and 66 controls. Student’s *t*-test revealed 195 significantly differential variables, with an error correction factor false discovery rate (FDR) < 0.05. After reviewing the data matrix, we selected the significant metabolites which were differentially increased or decreased by >30%. This difference was the variability assumed to be caused by the biological conditions of the individuals.

The multivariable analysis was performed using a supervised method, PLS-DA, taking into account the significant variables and those presenting a 1.3-fold change with respect to the group mean (Figure 3B).

As can be seen in Figure 3B, the groups presented good separation. Only two of the principal components were needed to explain more than 60% of the supervised model. Some outliers appeared and were eliminated. The analysis was then repeated, but this revealed further outliers. When these were also eliminated, the analysis was repeated, and the same result occurred. After reviewing the models obtained with each of these matrices, we decided to continue with the matrix that contained the largest sample size, as the results obtained with the different matrices of subjects did not vary greatly from the initial analysis.

**Figure 4:**
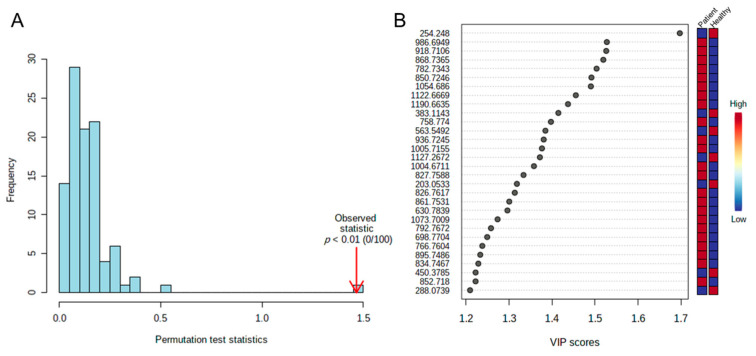
Quality and design of the suggested model. (**A**) Permutation test with 100 observations. (**B**) Graph representing the 30 variables with the best projection in the partial least squares regression analysis.

The fit of the model and its predictive capacity were measured by the R^2^ value. For three components, R^2^ = 0.57 and Q^2^ = 0.48. A permutation test based on 100 observations was then conducted (see Figure 4A), obtaining *p* < 0.01, which was significant for the multivariable model obtained.

The variables that contributed most to the model (i.e., those with a value >1) were selected, considering variable importance in projection (VIP) scores. Figure 4B shows the 30 variables with the best projections in the model.

### 2.3. Identifying Significant Variables

The next stage of analysis consisted of identifying the significant variables with FDR < 0.05 and with a 1.3-fold change with respect to the group mean. Most of the metabolites were identified assuming a mass error ≤5 ppm and by assigning a molecular formula using the formula finder tool. These findings were compared with the fragmentation spectrum of analytical standards obtained experimentally elsewhere, which are available for consultation in various public and private databases. Most of the structures were assigned to level 2 of the Schymanski classification [19], compared to an experimental mass/mass spectrum. For some molecules, such as eicosatetraenoic acid, the exact location of the double bonds could not be determined. Some of the signals identified corresponded to a single molecule, forming different adducts from the metabolite by various modes of ionization. Table 2 shows the 28 variables that were identified, which corresponded to 20 metabolites. These identities were assigned taking into account the retention time obtained in the present reverse chromatography analysis, together with the properties observed regarding the polarity of the metabolites. The significance value and the corrected value (obtained from Student’s *t*-test) were also considered, as well as the fold change, the molecular formula, the mass error for that formula, the adduct formed during the ionization of the molecule, and the structure tentatively assigned to the mass charge.

The main molecules identified correspond to well-known metabolites that can be found in biological plasma samples (chemical structure shown in Supplementary Table S2). In addition, some commonly used drugs were identified as differential, indicating that some of the children in the study were taking other medications, mainly antipyretics.

Most of the variables found to be significant presented poor chromatographic retention. Moreover, many molecules overlapped within the same retention time, suggesting they were polar in nature. In addition, many were likely related; this fact, together with the high molecular weight, made identification very difficult. A growing volume of structural information is becoming available, which can enable us to better identify the metabolites present in biological matrices; thus, our understanding of these questions is continuously being updated and reviewed, enabling new identifications to be assigned to metabolites that were previously unknown.

### 2.4. Analysis of Biological Pathways

**Figure 5:**
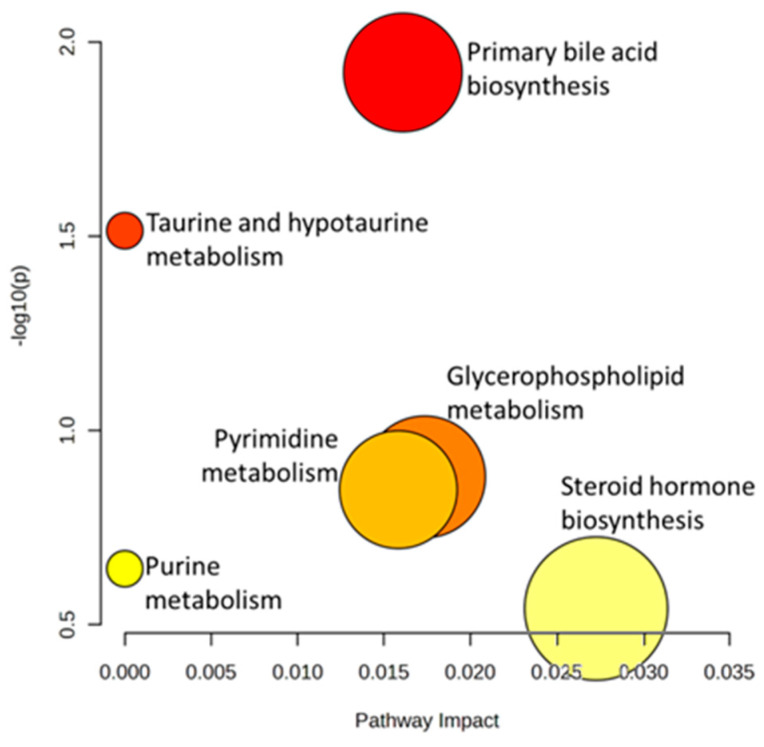
Summary of altered metabolic pathways analysis with MetaboAnalyst 5.0 reflecting the impact on the pathway and the level of significance. The colors of dots (varying from yellow to red) indicates the significance of the metabolites in the data. The size of the dot is positively correlated with the impact of the metabolic pathway.

Considering the metabolites identified, a pathway analysis was performed with MetaboAnalyst to determine which pathways might be altered and which presented significant differences. Figure 5 shows the main pathways in which controls and patients differ, together with the impact represented by the variation in these metabolites.

The pathways shown in more intense colors, such as red, correspond to higher levels of significance, while lighter colors represent less significance within the pathway. Another relevant consideration is the possible impact of the metabolite(s) on the pathway, represented by its involvement in different biotransformation processes within the same pathway; in other words, the more connections a given metabolite has in the production of others, the greater the impact on the pathway is due to the alteration of the first metabolite. Table 3 shows the *p*-values obtained for the pathways by each of the altered metabolites considered.

A significant outcome was the alteration in the biosynthesis of primary bile acids and the metabolism of taurine and hypotaurine. The production of primary bile acids is directly related to liver activity from cholesterol, and its binding to glycine and taurine serves to form bile salts, which contribute to facilitating the intestinal absorption of fats and fat-soluble vitamins.

### 2.5. Analysis of Biomarkers

**Figure 6:**
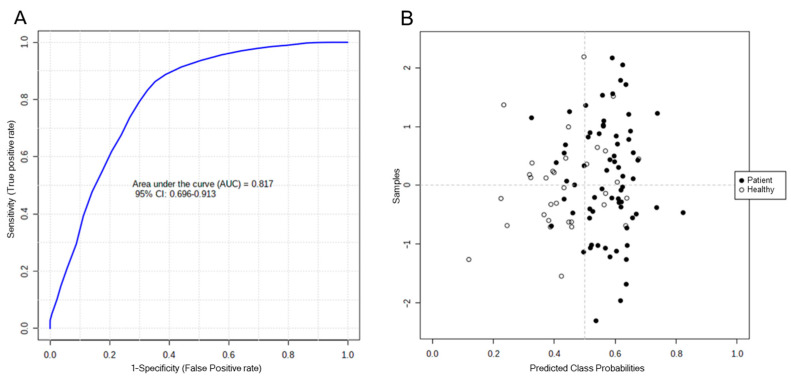
Model validation. (**A**) Area under the curve for the model with five tentatively identified metabolites (hydroxydecanoylcarnitine, octanoylcarnitine, lysophosphatidylcholine (16:1/0:0), glycocholic acid, and taurocholic acid). (**B**) Classification of individuals using a 100-fold cross-validation for each simple.

Biomarker analysis was performed using ROC curve analysis, combining some of the differential metabolites within a multivariate model, and focusing on those considered especially significant. The biomarker model sought was one that would allow us to classify and identify the children presenting hepatotoxicity, with good sensitivity and specificity. Accordingly, the following five metabolites were tentatively identified and combined; on the one hand, hydroxydecanoylcarnitine and octanoylcarnitine, with high values in the control group; on the other hand, lysophosphatidylcholine (16:1/0:0), glycocholic acid, and taurocholic acid, with high values in children with DILI. Figure 6A,B show the area under the curve for the model created with these five metabolites, using the PLS-DA algorithm, as well as the classifications obtained for the children included in each group.

The value obtained for the area under the curve was 0.817 (95% C.I: 0.696 to 0.913). The results based on cross-validation correctly classified 23 children with hepatotoxicity, but misclassified 10. In the control group, 56 children were classified correctly, and 12 were misclassified. Therefore, the model incorporating these five biomarkers could be used to identify hepatotoxicity in children. These results could also form the basis for seeking a model with better predictive capabilities, by including other metabolites, either already identified or pending identification.

## 3. Discussion

The untargeted metabolomic analysis performed on this group of children revealed clear differences between those who developed DILI and those who did not. The representation obtained using a multivariate model revealed significant differences in metabolomic composition between the two groups.

It is possible that drug-induced liver injury (DILI) in pediatrics differs substantially from adult DILI. The most frequently implicated drugs in the adult population are amoxicillin–clavulanate, flucloxacillin, atorvastatin, disulfiram, diclofenac, simvastatin, carbamazepine, ibuprofen, erythromycin, and anabolic steroids such as bodybuilding agents [20]. In the present study, the most frequently implicated drugs in DILI were antimicrobials. Another study conducted in hospitalized children in an intensive care unit found that medium- and long-chain lipid emulsions, sodium glycerophosphate, and meropenem were the most common drugs in the newborn, while omeprazole, methylprednisolone sodium succinate, and meropenem were the main culprits of DILI in children [5].

Primary bile acids, which are elevated in children with hepatotoxicity, could play a significant role in DILI. Taurine metabolism is another of the pathways found to be significantly altered.

To our knowledge, this study is the first to apply metabolomics to characterize idiosyncratic drug-induced liver injury in children. At present, hepatotoxicity is a diagnosis of exclusion that clinicians should suspect in patients with unexplained elevated liver enzymes. Therefore, new diagnostic and prognostic biomarkers are needed to achieve an early and reliable diagnosis of DILI and, thus, improve the prognosis. Although analytical, genetic, and pharmacokinetic approaches have obtained several DILI biomarkers, none provide sufficient specificity and sensitivity, and new approaches, such as the one described in this paper, are needed.

One of the greatest strengths of the present study is its use of the updated RUCAM method for diagnosis. From 1993 to mid-2020, RUCAM assessed a total of 95,865 cases of drug-induced liver injury (DILI) and herb-induced liver injury, surpassing any other causality assessment method in terms of case numbers. The success of RUCAM can be attributed to its quantitative characteristics with specific data elements that are individually scored, leading to a final causality assessment. RUCAM is objective, user-friendly, transparent, and specific to liver injuries; thus, it should be used in future cases of DILI [21].

In the present study, unfortunately, many molecules remained unidentified, due to the lack of experimental fragmentation spectra. Moreover, on some occasions, it was difficult to exactly assign a single molecular formula to a metabolite. On the other hand, our study was limited by the reduced number of samples for comparison, which was mainly due to the low number of pediatric patients diagnosed with DILI who allowed the use of samples for this type of study, in addition to the difficulty in obtaining samples for use as pediatric population controls.

Various animal studies and in vitro models have identified changes in the metabolomic profile in the presence of DILI [13,16,22,23,24,25,26,27,28,29,30,31,32,33,34,35].

The metabolomic profile has been studied in children, but only concerning intrinsic toxicity by paracetamol [36].

Adult humans are characterized by individual metabolic phenotypes [37,38,39,40,41]; there are areas of the metabolome unique to each individual that remain stable over time. “Metabolic profiles” can be detected that allow us to predict which individuals may present ways of metabolizing drugs that make them more susceptible to presenting DILI [17].

Winnike et al. [42], after a study carried out in adults analyzing metabolomic profiles in urine samples before and after receiving 4 g of paracetamol for 7 days (a regimen that produces mild liver damage in approximately one-third of the subjects), observed that the metabolomic profiles in urine prior to treatment were not sufficient to predict the development of mild liver damage, but those obtained after paracetamol administration did predict this effect. The authors concluded that, after drug administration, changes in urinary metabolites may determine which individuals will have only mild liver damage from those who are likely to develop more severe liver damage.

Elevated circulating levels of bile acids have been associated with major risk factors for non-alcoholic fatty liver disease (NAFLD), including insulin resistance and type 2 diabetes mellitus [43,44]. In addition, metabolomic studies conducted in the adult population have revealed global alterations in bile acid composition in individuals with simple steatosis compared with healthy controls [45,46]. Bile acids have also been studied as a potential therapeutic target in NAFLD [45,46,47], due to their signaling capabilities.

Xie et al. [48] identified 31 metabolites related to the severity of idiosyncratic DILI. Primary bile acid biosynthesis and alpha-linolenic acid metabolic pathways were also related to DILI severity.

Recent studies in adults showed that metabolomics can help establish markers of severity and/or chronicity of DILI. He et al. (May 2022) concluded in a study conducted in patients with DILI fibrosis that metabolomic fingerprints suggest that alteration of lipid metabolites is the most important factor in the development of DILI fibrosis [49]. Zhao et al. (April 2022), after performing a metabolomic study in patients with DILI, revealed that bile acids and polyunsaturated fatty acids were closely related to the severity and chronicity of DILI, respectively, thus potentially representing potent markers of DILI severity and chronicity [50].

Likewise, metabolomics not only is an effective tool for understanding the pathophysiology of DILI, but can also help predict the risk of liver damage [51].The bile acid metabolomic spectrum has also been postulated as an early biomarker of liver injury in children with infectious mononucleosis. Shen et al. (2023), in a case–control study with a total of 60 children with infectious mononucleosis (half of them with liver injury) and 30 healthy children, revealed statistically significant differences in serum bile acid spectrum before hepatic injury in children with infectious mononucleosis. It was concluded that the metabolomic analysis can sensitively detect the changes in serum bile acid spectrum before hepatic injury, which is helpful for early assessment of hepatic injury in children with infectious mononucleosis [52].

Levels of hydroxydecanoylcarnitine and octanoylcarnitine were elevated in the control group, apparently exerting a protective effect against DILI. Acetyl-L-carnitine is an effective substrate for mitochondrial energy metabolism. Carnitine also has free-radical-scavenging activity that enhances antioxidant status [53]. The hepatoprotective effects of carnitine against various agents, including acetaminophen, sodium valproate, and arsenic, have been proposed and demonstrated [53,54,55].

It has been suggested that carnitine administration could ameliorate NAFLD and non-alcoholic steatohepatitis [56,57]. These authors hypothesized that carnitine might lower liver enzymes by reducing beta-oxidation and limiting oxidative stress in the mitochondria, as well as modulating the inflammatory response. In this respect, Lheureux et al. also suggested that carnitine may protect the cell from the membrane-destabilizing effects of toxic acyl groups and, thus, prevent intramitochondrial accumulation [55]. The potential hepatoprotective effect of carnitine against drugs used to combat tuberculosis was demonstrated in a clinical study in which the authors suggested that carnitine could improve liver function by decreasing oxidative stress, increasing free-radical scavenging, improving mitochondrial function, and modulating lipid peroxidation [58].

The metabolomic analysis we described demonstrates the metabolic differences that arise once the hepatotoxic reaction has occurred; hence, its clinical significance may be even greater.

In this study, univariate analysis identified 148 significant metabolites with large expression differences. Of these metabolites, some were related to drug delivery at specific times without producing a harmful effect, but others, such as primary bile acids, participated directly in the metabolism of interest, provoking a direct effect on liver function. Subsequent analysis, focused on the metabolic pathways signaled by these metabolites, confirmed that the alteration of these metabolites significantly affected bile acid biosynthesis pathways.

The metabolites found to be differential may help us distinguish the molecular mechanisms that contribute to the appearance of hepatotoxicity, thus providing valuable insights into this disease among children. Some of the biliary metabolites may result as a consequence of liver damage following DILI as results of inhibition of bile salt export pump (BSEP). These metabolites could be a putative biomarker for prediction or confirmation of DILI, or even predict its triggering at an early stage through possible monitoring of blood levels. It is possible that the markers identified in pediatric DILI are not specific, and some of these markers may be shared with other hepatopathologies in the general population, necessitating future integrated studies in depth [59].

Further validation using an independent cohort is now required in order to confirm the classification capacity of the markers found, and to facilitate a targeted analysis, with the absolute quantification of primary and secondary bile acids. Success in these areas would improve our understanding of hepatic alterations in pediatric patients. The results obtained are an approximation of initial interest, with sample limitations, which represent a field of interest for further research on the metabolites of interest in future investigations, although there is still a long way to go before metabolomics can be transferred to the diagnosis and monitoring of DILI in clinical practice. Moreno-Torres et al. in a recent review (June 2022) recognized that the current diagnosis of drug-induced liver injury is based on determination of transaminases and serum bilirubin levels that are not specific for DILI; thus, there is growing interest in the discovery of new DILI-specific biomarkers. Metabolomics is a fundamental tool to detect potential biomarkers that allow the diagnosis of diseases, as well as an evaluation of the efficacy of drugs or their toxicity. Metabolomics has undoubtedly contributed to the understanding of the underlying mechanisms of DILI and has allowed the identification of metabolites as potential biomarkers, but there are still limitations preventing these research findings from being translated into general clinical practice, probably due to the variability of the methods used in the different studies, as well as due to the different mechanisms caused by the agent that causes or prevents DILI [60].

Since the metabolomic study was conducted once the probability diagnosis of DILI according to RUCAM was made, it is not possible to conclude that the alteration of bile acids is the consequence of liver alteration. Although this seems to be the most probable theory, one cannot rule out the possibility that it might be the cause determining the DILI and not its consequence.

DILI in children is a rare health issue. Due to this fact, the sample size of the present study did not allow differentiation between groups of drugs that act through the inhibition of BSEP and those that do so through other mechanisms. Classifying drugs according to their mechanism of action will further clarify the findings in future studies.

For this reason, we believe that more studies are necessary in children with a larger sample size that allows us to validate our results and help extrapolate the experimental results to clinical practice.

## 4. Materials and Methods

### 4.1. Study Population

The study population was composed of 143 pediatric patients, all aged between 0 and 15 years. Of these, 55 were diagnosed with DILI, and 88 (the controls) did not present liver toxicity. Moreover, 45 of these patients presented an oncological process, and 23 of the latter had high levels of transaminases. Data for the non-oncology study population are summarized in Table S1 of the Supplementary Materials. Table S1 details the epidemiological characteristics of the study population (cases and controls) such as sex, age, race, diagnosis, drug treatment, and RUCAM score of children with DILI reported for the time of sample analysis. 

The operational structure of the registry, the data recording process, and the characterization of cases were described in detail by Ocete Hita et al. (2013) [9]. These are summarized below.

(a) The criteria for diagnosing DILI among the patients at the time of their inclusion in the study include a chronological relationship between drug intake and the onset of hepatitis, and the presence of any of the following conditions: (1) level of alanine aminotransferase (ALT) more than five times the upper limit of normality (ULN); (2) level of alkaline phosphatase (ALP) more than double the ULN; (3) level of ALT more than three times the ULN, with concomitant elevation of bilirubin levels to more than double the ULN. The pattern of liver injury can be assessed by the R value, where R = (ALT/ULN)/(ALP/ULN); R ≥ 5 reflects a hepatocellular pattern, 2 < R < 5 reflects a mixed pattern, and R ≤ 2 reflects a cholestatic pattern [61].
(b) If one of the above red flags is detected, cases are reported using a structured protocol to exclude possible alternative causes. A detailed medical history is obtained from all patients regarding biliary or liver disease, as well as information on risk factors associated with liver disease. Serological markers of acute viral hepatitis are determined in all patients prior to diagnosis of DILI, together with serum ceruloplasmin and a battery of autoantibodies related to autoimmune liver disease.
(c) Causality is determined by the RUCAM evaluation method [61].
(d) In every case, a serum sample is extracted for the metabolomic study, at a time selected during the follow-up to coincide with the scheduled clinical control visits.

All patients were enrolled in the Spanish registry of hepatotoxic reactions among the pediatric population, which was created in 2008. The samples were collected and managed by the Biobank of the Public Health System of Andalusia in accordance with its internal procedures.

### 4.2. Ethics Approval and Consent to Participate

This study was conducted in accordance with the Declaration of Helsinki (as revised in 2013). Ethical approval for the study was obtained from the Granada Provincial Research Ethics Committee (Ethics permission number: 0057-M1-20). Informed patient consent was obtained for all samples used in this study.

### 4.3. Preparation of the Plasma Samples

The plasma samples were prepared for analysis from an aliquot with a volume of 100 μL. Protein precipitation was performed using an organic solvent; in this case, 800 μL of cold acetonitrile was added to promote metabolite extraction. After adding the solvent, the mixture was vortexed for 1 min at 2500 rpm, and the samples were centrifuged at 13,500 rpm for 15 min at 4 °C. After this step, a supernatant volume of 800 μL was collected and then evaporated in a centrifugal evaporator, for approximately two hours. The samples were then reconstituted in a volume of 250 μL in a water/acetonitrile (50/50) solution. Once all the samples had been reconstituted, the quality control samples were prepared from a 10 μL aliquot from each sample. In addition, a blank sample was prepared, using the same procedure as for the plasma samples, from PBS. This sample was used to eliminate signals that did not come from the plasma.

### 4.4. Analysis by Liquid Chromatography Coupled with High-Resolution Mass Spectrometry (HPLC–MS/MS)

Untargeted analysis was performed using an Agilent1290 chromatograph coupled to a QTOF5600 high-resolution mass spectrometer (SCIEX, RRID:SCR_018053, Agilent Technologies, Santa Clara, CA, USA).

The chromatographic method used included a mobile phase A consisting of water/acetonitrile (90/10) with 0.1% formic acid and a mobile phase B consisting of acetonitrile/water (90/10) and 0.1% formic acid. The chromatographic run was performed for 20 min with a C18 reverse-phase analytical column (Atlantis T3, Waters Corporation, Milford, MA, USA), with dimensions 150 × 2.1 mm, and a particle size of 3 μm. The column oven was set at a temperature of 20 °C. The chromatographic gradient is detailed in Table 4. The injection volume was 3 μL.

The untargeted analysis was carried out by positive electrospray ionization, with the following source parameters: temperature, 500 °C; gas 1 and gas 2, maintained at 50 psi; ionization spray voltage, 5000 V; curtain gas, 40 psi.

The collision energy was applied in a range of 15 to 45 V. The mass spectrometer was operated in the information-dependent acquisition mode, in which the eight most intense candidates were fragmented in each analysis cycle [62].

### 4.5. Data Matrix for Statistical Analysis and Analytical Validation

After performing the differential molecular identification, a signal matrix was obtained using MarkerView Software (SCIEX) version 1.3.1, with the following extraction parameters: min. retention time = 1.00 min; max. retention time = 16.00 min; subtraction offset = 10 scans; subtraction mult. factor = 1.3; noise threshold = 100 min. spectral peak width = 5 scans; retention time tolerance = 0.10 min; mass tolerance = 12.0 ppm; use global exclusion list = false; number of samples required = 30; max. number of peaks = 5000; use raw data area = true. 

Analytical validation was carried out using principal component analysis (PCA) to test the clustering of quality control samples injected during the analysis.

### 4.6. Statistical Analysis

The following statistical analyses were performed. In the initial univariate analysis, Student’s *t*-test was applied, normalizing from a quality control sample, and scaling and transforming the previously obtained data. Partial least squares discriminant analysis (PLS-DA) was then applied to determine a classification model that could incorporate a large number of variables.

### 4.7. Identifying Significant Variables

The signals found to be relevant in the univariate and multivariate analyses were then identified. A molecular formula was assigned, using the formula finder tool in PeakView software version 1.0 (RRID:SCR_015786) and consulting appropriate databases, including NIST 2.3 (RRID:SCR_006452), LIPID MAPS (RRID:SCR_006579), the Human Metabolome Database (RRID:SCR_007712), and MassBank (RRID:SCR_015535).

## 5. Conclusions

The approach we described for studying patients with drug-induced liver injury (DILI) is in accordance with similar studies considered innovative and might offer several advantages over current methods used for diagnosis and monitoring. Specifically, this strategy represents a more direct and specific diagnostic approach, enabling precise monitoring of DILI and determining the degree of functional recovery of the affected liver, beyond the mere absence of enzyme markers in the blood. The accumulation of intrahepatic cytotoxic bile acids due to drug-induced alterations in bile acid homeostasis could be an important factor contributing to the development of DILI in susceptible children. Therefore, analyzing the circulating concentrations of individual bile acids could provide early, sensitive, and selective markers for monitoring the functional decline of the liver.

## Figures and Tables

**Table 1:** Summary of clinical characteristics of the 32 children with DILI: type of liver injury, clinical presentation, most frequent symptoms, median days from the start of drug treatment to the onset of symptoms, and median days of drug treatment. DILI, drug-induced liver injury.

Clinical Characteristics	n	%	Days (Median ± SD)
Type of injury			
Hepatocellular injury	29	90.63	
Cholestatic and mixed injury	3	9.38	
Clinical presentation			
Fulminant course (liver transplant, death)	2	6.25	
Severe injury	5	15.63	
Chronicity	1	3.13	
Clinical signs and symptoms			
Abdominal pain	25	78.13	
Nausea, vomiting	22	68.75	
Anorexia, asthenia	19	59.38	
Days from drug start to onset of symptoms			8 ± 3
Total days of drug use			5 ± 6

**Table 2:** Characteristics of the significant differential variables between the control group and the group of children with DILI. The *p*-value was calculated using Student’s *t*-test. * FDR: false discovery rate; Δppm: deviation of the measured mass from the theoretical mass in parts per million.

Mass/ Charge	*p*-Value	FDR *	Retention Time	Fold Change	Log (Fold Change)	Molecular Formula	Δppm	Structure	Adduct
152.0695	2.6 × 10^−3^	6.5 × 10^−3^	3.08	13.243	1.122	C8H9NO2	−4.0	Acetaminophen	H
203.0533	1.2 × 10^−6^	1.5 × 10^−5^	1.33	1.554	0.191	C6H12O6	1.4	Glucose	Na
383.1143	2.3 × 10^−7^	7.5 × 10^−6^	1.32	1.643	0.216	C6H12O6			2M + Na
205.1227	1.9 × 10^−3^	4.9 × 10^−3^	6.59	4.187	0.622	C13H16O2	0.5	Hydroxy-ibuprofen	H-H20
218.1282	1.3 × 10^−2^	2.6 × 10^−2^	2.26	0.127	−0.897	C12H15N3O	1.0	Noramidopyrine	H
245.0742	1.6 × 10^−6^	1.7 × 10^−5^	1.36	1.608	0.206	C9H12N2O6	7.8	Uridine	H
254.248	1.8 × 10^−8^	1.6 × 10^−6^	13.14	2.009	0.303	C16H31NO	0.6	Palmitoleamide	H
265.2514	1.6 × 10^−8^	1.6 × 10^−6^	15.27	1.764	0.247	C18H32O	−4.2	9-Octadecenamide	H-NH3
282.2787	1.5 × 10^−8^	1.6 × 10^−6^	15.27	1.824	0.261	C18H35NO	0.6		H
563.5492	1.1 × 10^−7^	5.8 × 10^−6^	15.3	2.269	0.356	C36H70N2O2	−1.8		2M + H
280.2641	4.5 × 10^−7^	9.5 × 10^−6^	13.63	1.744	0.242	C18H33NO	0.4	Linoleic acid conjugate	H
288.2169	5.2 × 10^−3^	1.2 × 10^−2^	5.81	1.515	0.180	C15H29NO4	0.2	O-octanoylcarnitine	H
305.2464	1.4 × 10^−3^	3.9 × 10^−3^	15.03	1.449	0.161	C20H32O2	1.6	cis-5,8,11,14-eicosatetraenoic acid	H
317.0541	9.3 × 10^−3^	1.9 × 10^−2^	1.41	4.143	0.617	C10H12N4O6	2.7	Xanthosine	
320.256	7.0 × 10^−5^	2.9 × 10^−4^	10.71	1.364	0.135	C17H36O5	−0.7	Tetraethylene glycol	Na
320.2575	2.8 × 10^−5^	1.5 × 10^−4^	11.01	1.377	0.139	C18H35NO2	1.6	3-ketosphingosine	H
328.1383	3.9 × 10^−4^	1.3 × 10^−3^	1.79	1.479	0.170	C15H21NO7	0.4	N-(1-Deoxy−1-fructosyl)phenylalanine	H
329.2467	1.4 × 10^−3^	3.8 × 10^−3^	14.56	1.342	0.128	C22H32O2	0.0	Docosapentaenoic acid (22n-6)	H
332.2425	1.8 × 10^−4^	7.0 × 10^−4^	5.81	1.333	0.125	C17H33NO5	−0.8	Hydroxidecanoylcarnitine	H
363.2164	8.2 × 10^−4^	2.6 × 10^−3^	6.58	1.441	0.159	C21H30O5	0.2	Cortisol	H
412.283	5.0 × 10^−3^	1.1 × 10^−2^	7.37	0.119	−0.924	C26H37NO3	0.4	Glycocholic acid	H-3H20
430.2934	5.5 × 10^−3^	1.2 × 10^−2^	7.37	0.124	−0.908	C26H39NO4	−0.2		H-2H20
448.3064	5.2 × 10^−3^	1.2 × 10^−2^	7.37	0.118	−0.928	C26H41NO5	0.3		H-H20
466.3168	8.1 × 10^−3^	1.7 × 10^−2^	7.37	0.113	−0.946	C26H43NO6	0.2		H
488.2989	4.2 × 10^−3^	9.5 × 10^−3^	7.37	0.181	−0.743	C26H43NO6	−1.1		Na
494.3248	7.7 × 10^−3^	1.6 × 10^−2^	9.96	0.485	−0.314	C24H48NO7P	0.8	Phosphatidylcholine (16:1/0:0)	H
516.2975	4.1 × 10^−3^	9.3 × 10^−3^	7.9	0.107	−0.971	C26H45NO7S	−0.1	Taurocholic acid	H
533.3235	4.0 × 10^−3^	9.3 × 10^−3^	7.94	0.127	−0.895	C26H45NO7S	−0.4		NH4

**Table 3:** Main pathways that may be altered and their significance (*p*-value) in relation to the differential metabolites between the groups.

Pathway Name	Total Metabolites in the Pathway	Common	*p*-Value	Impact on the Pathway
Biosynthesis of primary bile acids	46	2	0.0271	0.0161
Metabolism of taurine and hypotaurine	8	1	0.04562	0.0000
Metabolism of glycerophospholipids	36	1	0.19108	0.0174
Metabolism of pyridines	39	1	0.20543	0.0158
Metabolism of purines	65	1	0.32062	0.0000
Biosynthesis of steroid hormones	85	1	0.39887	0.0273

**Table 4:** Chromatographic gradient analysis with reverse chromatography and positive ionization.

Time (min)	Flow (μL/min)	% Mobile Phase A	% Mobile Phase B
0.0	300	99	1
0.5	300	99	1
11.0	300	1	99
15.5	300	1	99
15.6	300	99	1
20.0	300	99	1

## Data Availability

The analyzed data of the present study are available to any researcher upon request to any of the authors.

## Supplementary Materials

The following supporting information can be downloaded at https://www.mdpi.com/article/10.3390/ijms241713562/s1.
