# Supplementary material for: Metabolomic Analysis of Pediatric Patients with Idiosyncratic Drug-Induced Liver Injury According to the Updated RUCAM
Source: Int J Mol Sci. 2023 Sep 1;24(17):13562. doi: 10.3390/ijms241713562 (PMC10487599; doi:10.3390/ijms241713562)
Supplement: Supplementary file 1 [file ijms-24-13562-s001.zip › ijms-2556125-supplementary.pdf]

# Supplementary Material: Table S1 and S2

- Table S1: Epidemiological characteristics of 32 children with DILI and 66 children without DILI: sex, age, race. Diagnosis, drug treatment and RUMCAM score of children with DILI reported for the time of sample analysis.

| SEX | AGE (years) | RACE | DILI/ Without DILI | DIAGNOSIS | DRUG | RUCAM SCORE |
|---|---|---|---|---|---|---|
| Male | 5 | Caucasian | DILI | Fever | Ibuprofen | 9 |
| Female | 1 | Caucasian | DILI | Pneumococcal meningitis | Rifampicin | 7 |
| Female | 2 | Caucasian | DILI | Systemic inflammatory response syndrome | Meropenem | 7 |
| Female | 2 | Caucasian | DILI | Sepsis | Meropenem | 6 |
| Female | 3 | Caucasian | DILI | Reunatoid arthritis | Methotrexate | 5 |
| Male | 1 | Caucasian | DILI | Tachyarrhythmia | Amiodarone | 8 |
| Male | 1 | Caucasian | DILI | Cardiomyopathy | Propranolol | 7 |
| Male | 0,5 | Caucasian | DILI | Sepsis | Meropenem | 6 |
| Male | 1 | Caucasian | DILI | Sepsis | Meropenem | 4 |
| Female | 1 | Caucasian | DILI | Pneumonia (Candida) | Voriconazole | 7 |
| Female | 3 | Caucasian | DILI | Seizures | Zonisamide | 8 |
| Female | 3 | Caucasian | DILI | Sepsis (Candida) | Voriconazole | 6 |
| Male | 5 | Caucasian | DILI | Sepsis (S.aureus) | Cloxacillin | 7 |
| Female | 10 | Caucasian | DILI | Tonsillitis | Amoxicillin | 7 |
| Male | 15 | Caucasian | DILI | Ataxia-teleangiectasia | Cotrimoxazole | 6 |
| Female | 14 | Caucasian | DILI | Encephalitis | Acyclovir | 5 |
| Female | 5 | Caucasian | DILI | Pulmonary tuberculosis | Isoniacid | 8 |
| Female | 3 | Caucasian | DILI | Hip synovitis | Ibuprofen | 6 |
| Female | 3 | Caucasian | DILI | Hip synovitis | Ibuprofen | 5 |
| Female | 1,5 | Caucasian | DILI | Juvenile idiopathic arthritis | Methotrexate | 4 |
| Male | 5 | Caucasian | DILI | Respiratory infection | Amoxicillin-clavulanic acid | 6 |
| Male | 9 | Caucasian | DILI | Bronchitis | Amoxicillin-clavulanic acid | 7 |
| Female | 1 | Caucasian | DILI | Respiratory infection | Amoxicillin-clavulanic acid | 5 |
| Male | 0,5 | Caucasian | DILI | Respiratory infection | Amoxicillin-clavulanic acid | 4 |
| Male | 1 | Caucasian | DILI | Pultaceous tonsillitis | Amoxicillin-clavulanic acid | 5 |
| Male | 14 | Caucasian | DILI | Tonsillitis | Amoxicillin-clavulanic acid | 7 |
| Male | 5 | Caucasian | DILI | Mastoiditis | Amoxicillin-clavulanic acid | 6 |
| Male | 0,5 | Caucasian | DILI | Respiratory infection | Amoxicillin-clavulanic acid | 5 |
| Male | 10 | Caucasian | DILI | Respiratory infection | Amoxicillin-clavulanic acid | 5 |
| Male | 3,5 | Caucasian | DILI | Vomiting | Green anise | 6 |
| Male | 4 | Caucasian | DILI | Vomiting | Green anise | 8 |
| Male | 0,2 | Caucasian | DILI | Aerocolia | Horsetail (Equisetum) | 4 |
| Female | 5 | Caucasian | Without DILI | NA | NA | NA |
| Female | 3 | Caucasian | Without DILI | NA | NA | NA |
| Male | 5 | Caucasian | Without DILI | NA | NA | NA |
| Female | 6 | Caucasian | Without DILI | NA | NA | NA |
| Female | 6 | Caucasian | Without DILI | NA | NA | NA |

| SEX | AGE (years) | RACE | DILI/ Without DILI | DIAGNOSIS | DRUG | RUCAM SCORE |
|---|---|---|---|---|---|---|
| Female | 8 | Caucasian | Without DILI | NA | NA | NA |
| Male | 9 | African | Without DILI | NA | NA | NA |
| Male | 4 | Caucasian | Without DILI | NA | NA | NA |
| Male | 4 | Caucasian | Without DILI | NA | NA | NA |
| Female | 3 | Caucasian | Without DILI | NA | NA | NA |
| Female | 0,5 | Caucasian | Without DILI | NA | NA | NA |
| Male | 1 | Caucasian | Without DILI | NA | NA | NA |
| Male | 2 | Caucasian | Without DILI | NA | NA | NA |
| Female | 1 | African | Without DILI | NA | NA | NA |
| Male | 1 | Caucasian | Without DILI | NA | NA | NA |
| Female | 5 | Caucasian | Without DILI | NA | NA | NA |
| Male | 4 | Caucasian | Without DILI | NA | NA | NA |
| Male | 2 | Caucasian | Without DILI | NA | NA | NA |
| Male | 3 | Caucasian | Without DILI | NA | NA | NA |
| Male | 2 | Caucasian | Without DILI | NA | NA | NA |
| Male | 7 | Caucasian | Without DILI | NA | NA | NA |
| Female | 5 | Caucasian | Without DILI | NA | NA | NA |
| Female | 9 | Caucasian | Without DILI | NA | NA | NA |
| Male | 12 | Caucasian | Without DILI | NA | NA | NA |
| Female | 3 | Caucasian | Without DILI | NA | NA | NA |
| Male | 2 | Caucasian | Without DILI | NA | NA | NA |
| Male | 13 | Caucasian | Without DILI | NA | NA | NA |
| Male | 11 | Caucasian | Without DILI | NA | NA | NA |
| Male | 3 | African | Without DILI | NA | NA | NA |
| Male | 2 | Caucasian | Without DILI | NA | NA | NA |
| Female | 4 | Caucasian | Without DILI | NA | NA | NA |
| Male | 5 | Caucasian | Without DILI | NA | NA | NA |
| Female | 2 | Caucasian | Without DILI | NA | NA | NA |
| Male | 1 | Caucasian | Without DILI | NA | NA | NA |
| Female | 7 | Caucasian | Without DILI | NA | NA | NA |
| Male | 8 | Caucasian | Without DILI | NA | NA | NA |
| Male | 10 | Caucasian | Without DILI | NA | NA | NA |
| Female | 2 | Caucasian | Without DILI | NA | NA | NA |
| Male | 1 | Caucasian | Without DILI | NA | NA | NA |
| Female | 3 | Caucasian | Without DILI | NA | NA | NA |
| Male | 1 | Caucasian | Without DILI | NA | NA | NA |
| Male | 2 | Caucasian | Without DILI | NA | NA | NA |
| Male | 4 | Caucasian | Without DILI | NA | NA | NA |
| Male | 4 | Caucasian | Without DILI | NA | NA | NA |
| Male | 5 | Caucasian | Without DILI | NA | NA | NA |
| SEX | AGE (years) | RACE | DILI/ Without DILI | DIAGNOSIS | DRUG | RUCAM SCORE |

| | | | | | | |
|---|---|---|---|---|---|---|
| Male | 6 | Caucasian | Without DILI | NA | NA | NA |
| Female | 2 | Caucasian | Without DILI | NA | NA | NA |
| Male | 6 | Caucasian | Without DILI | NA | NA | NA |
| Male | 10 | African | Without DILI | NA | NA | NA |
| Male | 12 | Caucasian | Without DILI | NA | NA | NA |
| Female | 13 | Caucasian | Without DILI | NA | NA | NA |
| Female | 2 | Caucasian | Without DILI | NA | NA | NA |
| Female | 1 | Caucasian | Without DILI | NA | NA | NA |
| Female | 2 | Caucasian | Without DILI | NA | NA | NA |
| Female | 1 | Caucasian | Without DILI | NA | NA | NA |
| Female | 2 | Caucasian | Without DILI | NA | NA | NA |
| Female | 4 | Caucasian | Without DILI | NA | NA | NA |
| Male | 3 | Caucasian | Without DILI | NA | NA | NA |
| Male | 6 | Caucasian | Without DILI | NA | NA | NA |
| Female | 2 | Caucasian | Without DILI | NA | NA | NA |
| Female | 4 | African | Without DILI | NA | NA | NA |
| Male | 3 | Caucasian | Without DILI | NA | NA | NA |
| Male | 6 | Caucasian | Without DILI | NA | NA | NA |
| Male | 7 | Caucasian | Without DILI | NA | NA | NA |
| Female | 2 | Caucasian | Without DILI | NA | NA | NA |
| Female | 1 | Caucasian | Without DILI | NA | NA | NA |

Abbreviations: NA = Not aplicable.

**Table S2.-** Chemical structure of the main metabolites identified in DILI samples patients.

| **Metabolite** | **Chemical structure** |
|---|---|
| Hydroxydecanoylcarnitine | 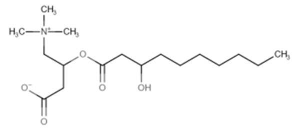 |
| Octanoylcarnitine | 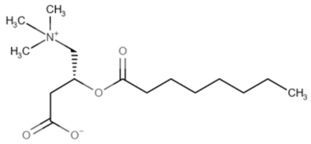 |
| Lysophosphatidylcholine | 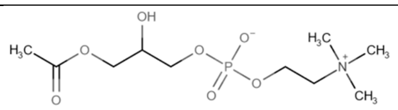 |
| Glycocholic acid | 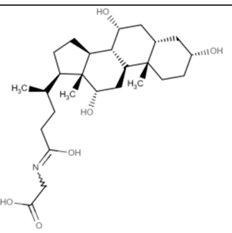 |
| Taurocholic acid | 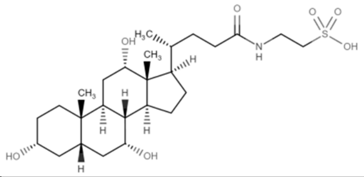 |
